# Ezetimibe Normalizes Dietary Cholesterol-Induced Exacerbation of Liver Injury in Alcohol-Fed Mice

**DOI:** 10.3390/biom16040590

**Published:** 2026-04-16

**Authors:** Yanchao Xu, Nan Zhang, Piumi B. Wickramasinghe, Kavya Veera, Preethi Parupalli, Alex Dao, Junyu Liu, Rithika Anand, Lyndsey E. Langley, Sreeja Eadha, Hasan Iqbal, Chen Liu, Fang Bian, Lin Jia

**Affiliations:** 1 Department of Molecular Genetics, University of Texas Southwestern Medical Center, Dallas, TX 75390, USA; yanchao.xu@utsouthwestern.edu; 2 Department of Biological Sciences, The University of Texas at Dallas, Richardson, TX 75080, USA; nan.zhang@utdallas.edu (N.Z.); piumi.wickramasinghe@utdallas.edu (P.B.W.); kavya.veera@utdallas.edu (K.V.); preethi.parupalli@utdallas.edu (P.P.); thuong.dao@utdallas.edu (A.D.); junyu.liu@utdallas.edu (J.L.); rithika.anand@utdallas.edu (R.A.);; 3 Center for Hypothalamic Research, Department of Internal Medicine, University of Texas Southwestern Medical Center, Dallas, TX 75390, USA; 4 Department of Bioengineering, The University of Texas at Dallas, Richardson, TX 75080, USA; fang.bian@utdallas.edu; 5 Office of Research and Innovation, The University of Texas at Dallas, Richardson, TX 75080, USA

**Keywords:** dietary cholesterol, ezetimibe, ALD, fatty liver, cholesterol biosynthesis, biliary bile acid

## Abstract

Interactions between alcohol and nutrition play an important role in the development and progression of alcohol-associated liver disease (ALD). Although dietary cholesterol was shown to exacerbate fatty liver and liver injury in alcohol-fed mice, findings regarding the combined effect of dietary cholesterol and heavy alcohol drinking on cholesterol homeostasis remain controversial. Ezetimibe has been widely used as a cholesterol-lowering drug in hypercholesterolemic subjects. It is not fully understood whether ezetimibe blunts the adverse effect of cholesterol on lipid and biliary bile acid metabolism in alcohol-exposed mice. In the current study, wild-type mice were subjected to NIAAA alcohol feeding model. Dietary cholesterol (0.2%, *w*/*v*) and ezetimibe (0.001%, *w*/*v*) were added to the liquid diets. Cholesterol and triglyceride contents in the liver and circulation were determined. Biliary bile acid composition, as well as hepatic and circulating inflammatory markers were analyzed. We found that ezetimibe protected mice from the synergistic effects of dietary cholesterol and alcohol on hepatic triglyceride accumulation, which was accompanied by enhanced expression of genes involved in hepatic beta oxidation. Dietary cholesterol caused great increases in liver cholesterol content and dramatic reductions in the expression of hepatic cholesterol biosynthetic genes in both control- and alcohol-fed mice. These changes were normalized by ezetimibe treatment. Ezetimibe attenuated dietary cholesterol-induced elevations in total biliary bile acids. Moreover, mice fed a diet containing both cholesterol and alcohol exhibited increased expression of monocyte chemoattractant protein 1 (Mcp1) and tumor necrosis factor alpha (Tnfα) in the distal small intestine. Collectively, our findings indicate that ezetimibe effectively mitigates the adverse effects of dietary cholesterol and alcohol consumption on hepatic lipid accumulation and liver injury.

## 1. Introduction

Alcohol abuse is one of the major causes of chronic liver disease and liver-related death worldwide. Alcohol-associated liver disease (ALD) comprises a spectrum of pathological abnormalities. Although more than 90% of heavy drinkers develop fatty livers, approximately 10% to 40% of individuals with simple steatosis progress to steatohepatitis. In addition, among patients with alcoholic hepatitis, about 70% of them suffer from cirrhosis, a condition associated with high mortality [1]. These epidemiological findings suggest that in addition to alcohol itself, other factors contribute to disease progression. Recent studies have shown that dietary nutrients can significantly modulate alcohol-induced organ damage [2]. Specifically, a diet containing high cholesterol and alcohol synergistically induces more severe liver injury in rodents, with the development of hepatic inflammation and early fibrosis [3,4]. However, these studies incorporated 0.5% (*w*/*v*) cholesterol into alcohol containing diets [3,4], a level corresponding to an estimated human intake of approximately 500 mg/day. Of note, Bosner et al. reported that the average dietary cholesterol intake in US adults is approximately 300 mg/day [5]. In addition, the cholesterol absorption rate in C57BL/6 mice is typically 60–80% [6], which is higher than those observed in human subjects [7]. Therefore, it is necessary to perform rodent experiments using a lower cholesterol-containing diet, which is more relevant to human disease condition.

Dietary cholesterol has been shown to suppress endogenous cholesterol synthesis and promote hepatic cholesterol accumulation [8]. However, Li et al. reported that mice exposed to diets containing cholesterol and alcohol exhibited elevated hepatic expression of sterol regulatory element-binding protein 2 (SREBP2) and 3-hydroxy-3-methylglutaryl-coenzyme A reductase (HMGCR) [4]. Bile acids play important roles in maintaining whole-body cholesterol homeostasis. Bile acids are synthesized in the liver using cholesterol as a precursor. This synthetic pathway is one of the catabolic mechanisms contributing to cholesterol removal out of the body. Synthesized bile acids can be stored in the gallbladder and secreted into the gut, which are essential for intestinal cholesterol absorption. Dietary cholesterol either has no effect on or increases total biliary bile acid content [9,10]. Several studies reported that alcohol alone could influence total amount and composition of biliary bile acids [11,12]. However, the combined effects of dietary cholesterol and heavy alcohol drinking on gallbladder bile acids remain unclear.

Ezetimibe is a well-known cholesterol-lowering drug and has been used to treat hypercholesterolemia by blocking intestinal cholesterol uptake [13]. Moreover, several clinical trials suggest a protective role of ezetimibe in nonalcoholic fatty liver disease [14,15,16,17]. However, the potential role of ezetimibe in mitigating liver damage induced by concurrent intake of cholesterol and alcohol remains largely unexplored.

In the current study, 0.2% cholesterol (Chol, *w*/*v*) was added to Control- or EtOH-containing liquid diet (Control+Chol and EtOH+Chol diets). This dietary cholesterol content mimics the amount of daily average cholesterol consumption in US population and has been widely used in experimental animals [18,19,20,21]. To explore whether ezetimibe protects mice from cholesterol-induced perturbation of lipid and biliary bile acid metabolism, 0.001% ezetimibe (*w*/*v*) was supplemented to cholesterol-containing liquid diets (Control+Chol+Eze and EtOH+Chol+Eze). Mice were exposed to one of the six liquid diets by following the NIAAA (acute-on-chronic) alcohol feeding model. Our findings provided strong experimental evidence supporting ezetimibe as a potential therapeutic approach to attenuate liver injury in heavy drinkers consuming a high-cholesterol diet.

## 2. Materials and Methods

### 2.1. Animal Care and Diets

Male C57BL/6J mice (8 to 10 weeks old, Jackson Laboratory) were first subjected to the Lieber-DeCarli control liquid diet (F1259SP, Bio-Serv, Flemington, NJ, USA) for 5 days and then randomly divided into six groups for one of the following liquid diets for 14 days: (1) a control liquid diet (F1259SP, Bio-Serv; Control), (2) an alcohol-containing liquid diet (5% ethanol; F1258SP, Bio-Serv; EtOH), (3) a control liquid diet plus 0.2% cholesterol (*w*/*v*, MP Biomedicals) (Control+Chol), (4) an alcohol-containing liquid diet plus 0.2% cholesterol (*w*/*v*) (EtOH+Chol), (5) a control liquid diet plus both 0.2% cholesterol and ezetimibe (0.001%, *w*/*v*, in liquid diet, Sigma) (Control+Chol+Eze), (6) an alcohol-containing liquid diet plus both 0.2% cholesterol and ezetimibe (EtOH+Chol+Eze). This concentration of ezetimibe was calculated based on publications [22,23,24] and our own observation that a 20~25 g mouse consumes 10~13 mL of liquid diet per day and 0.005% (*w*/*w*, in pellet diet) ezetimibe effectively inhibits intestinal cholesterol absorption in mice [22,23,24]. Pair feeding was performed in Control diet-fed mice to match the liquid diet volume consumed by EtOH diet-fed mice. On the last day of liquid diet feeding, Control diet- and EtOH diet-fed mice were administrated with 9 g/kg maltose dextrin (45%, *w*/*v*) and 5 g/kg ethanol (31.5%, *v*/*v*) by oral gavage, respectively [25]. 6 h later, mice were euthanized for blood and tissue collections. Body weights and liver weights were recorded. Experiments were performed according to the protocol (Approval code: #20-10 and #2023-0122; Approval date: 22 October 2020 and 22 January 2023), reviewed and approved by the Institutional Animal Care and Use Committee of The University of Texas at Dallas (UTD). Mice were maintained in a temperature-controlled facility on a 12/12 h light/dark cycle.

### 2.2. Biochemical Assays

Blood was collected from inferior vena cava and transferred to EDTA-coated tubes. Plasma was separated by centrifugation at 12,000× *g* for 15 min and stored at −80 °C. Plasma alanine transaminase (ALT) and aspartate aminotransferase (AST) were measured using the Vitros 250 Chemistry Analyzer (Ortho Clinical Diagnostics, San Diego, CA, USA) at the UT Southwestern Metabolic Phenotyping Core. Plasma triglyceride, total cholesterol and free cholesterol were analyzed by enzymatic assay kits from Thermo Scientific™ (TR22421 and TR13421, Waltham, MA, USA) and Fujifilm (Lexington, MA, USA), respectively. Plasma concentrations of cholesterol ester were calculated by subtracting free cholesterol from total cholesterol. Plasma non-esterified fatty acid (NEFA) contents were determined by Wako NEFA Assay Kit (Fujifilm, Lexington, MA, USA). Plasma concentrations of total bile acids were determined using the Total Bile Acid (TBA) Colorimetric Assay Kit (BQ 092A-EALD, Bio-Quant, San Diego, CA, USA).

### 2.3. Measurement of Liver Lipid Contents

Frozen liver tissues (50–80 mg) were used for lipid extraction as described previously [26]. The corresponding standards for triglyceride (Verichem Laboratories, Providence, RI, USA), total cholesterol (Verichem Laboratories) and free cholesterol (Fujifilm) were prepared by adding 1% Triton X-100 in chloroform, evaporating, and dissolving in deionized water. Hepatic contents of triglyceride, total cholesterol and free cholesterol were quantified using the commercially available kits from Thermo Scientific™ (TR22421 and TR13421) and Fujifilm, respectively. Cholesterol ester was calculated as the difference between total and free cholesterol.

### 2.4. Tissue Histology

Liver tissues were collected and fixed in buffered 10% formalin and processed for hematoxylin and eosin (H&E) staining by the Histo Pathology Core at UT Southwestern Medical Center. The images were visualized and captured with a Zeiss microscope (Imager ZI, Zeiss, White Plains, NY, USA) equipped with a digital camera (Axiocam, Zeiss, White Plains, NY, USA).

### 2.5. Hepatic Contents of Lipid Peroxidation

Liver tissues (25–40 mg) were homogenized in a RIPA buffer (Cayman Chemical, Ann Arbor, MI, USA) and centrifuged at 1600× *g* for 10 min at 4 °C. Supernatant was collected for analysis of malondialdehyde (MDA), a naturally occurring product of lipid peroxidation, which were measured as thiobarbituric acid reactive substrates (TBARS) using the TBARS Assay Kit (Cayman Chemical, Ann Arbor, MI, USA).

### 2.6. Measurement of Fecal Neutral Sterol Excretion

After being fed the liquid diets for 11 days, mice were individually housed for 3 days for feces collection and provided with the same liquid diets. The feces were dried in a 70 °C vacuum oven for weight recording. The feces from each mouse were crushed into power. A total of 30~60 mg of feces was placed into a glass tube containing 100 µg of 5α-cholestane as an internal standard. After adding 2 mL of 95% ethanol and 200 µL 50% KOH, the feces were saponified in heating blocks at 70 °C for 3 h. Then the lipids were extracted by mixing with 2 ml of hexane and 2 ml of water and analyzed by gas-liquid chromatography in the MASS SPEC CORE at UTD.

### 2.7. Quantitative Real-Time PCR

Total RNAs from the liver and small intestine were isolated using RNA STAT60 (Tel-Test, Friendswood, TX, USA). Genomic DNAs were removed with a DNA removal kit (Invitrogen, Waltham, MA, USA) followed by cDNA synthesis using the High Capacity cDNA Kit (Applied Biosystems, Waltham, MA, USA) and iScript Advanced cDNA Synthesis Kit (Bio-Rad, Hercules, CA, USA). qPCR was performed in 384-well plates using CFX Opus 384 Real-Time PCR System (Bio-Rad, Hercules, CA, USA). Primers for CD36 (forward, GGAACTGTGGGCTCATTGC and reverse, CATGAGAATGCCTCCAAACAC), CPT1α (forward, CACCAACGGGCTCATCTTCTA and reverse, CAAAATGACCTAGCCTTCTATCGAA), Acox1 (forward, AGATTGGTAGAAATTGCTGCAAAA and reverse, ACGCCACTTCCTTGCTCTTC), SREBP1c (forward, GGGAGGACCCAAGGTGACA and reverse, CACGGACGGGTACATCTTTAAAG, FASN (forward, GCTGCGGAAACTTCAGGAAAT and reverse, AGAGACGTGTCACTCCTGGACTT), SS (forward, CCAACTCAATGGGTCTGTTCCT and reverse, TGGCTTAGCAAAGTCTTCCAACT), HMGCR (forward, CTTGTGGAATGCCTTGTGATTG and reverse, AGCCGAAGCAGCACATGAT), ABCA1 (forward, CGTTTCCGGGAGGTGTCCTA and reverse, GCTAGAGATGACAAGGAGGATGGA), LDLR (forward, AGGCTGTGGGCTCCATAGG and reverse, TGCGGTCCAGGGTCATCT), PCSK9 (forward, CAGGCGGCCAGTGTCTATG and reverse, GCTCCTTGATTTTGCATTCCA), NPC1L1 (forward, TGGACTGGAAGGACCATTTCC and reverse, GCGCCCCGTAGTCAGCTAT), PGC1α (forward, AACCACACCCACAGGATCAGA and reverse, CTCTCGCTTTATTGCTCCATGA), Nrf2 (forward, CCGCTACACCGACTACGATT and reverse, ACCTTCATCACCAACCCAAG), Cox2 (forward, GCCGACTAAATCAAGCAACA and reverse, CAATGGGCATAAAGCTATGG), Cyp7a1 (forward, AGCAACTAAACAACCTGCCAGTACTA and reverse, GTCCGGATATTCAAGGATGCA), Cyp8b1 (forward, GCCTTCAAGTATGATCGGTTCCT and reverse, GATCTTCTTGCCCGACTTGTAGA), Cyp27a1 (forward, GGAGGGCAAGTACCCAATAAGA and reverse, TGCGATGAAGATCCCATAGGT), Mcp1 (forward, TTTTTGTCACCAAGCTCAAGAGA and reverse, ATTTGGTTCCGATCCAGGTT), Tnfα (forward, CTGAGGTCAATCTGCCCAAGTAC and reverse, CTTCACAGAGCAATGACTCCAAAG), iNOS (forward, CAGGAGGAGAGAGATCCGATTTA and reverse, GCATTAGCATGGAAGCAAAGA) and 18s (forward, ACCGCAGCTAGGAATAATGGA and reverse, GCCTCAGTTCCGAAAACCA) were purchased from Integrated DNA Technologies.

### 2.8. Western Blotting

Livers were homogenized in lysis buffer containing 1% NP-40, 1% Triton-X 100, 1% SDS, 5 mM EDTA (pH 8.0), 50 mM Tris– HCl (pH 7.4), and protease inhibitor (P8340, Sigma, St. Louis, MO, USA) and phosphatase inhibitor cocktails (P5726 and P0044, Sigma, St. Louis, MO, USA). Protein concentrations were determined by BCA kit (Pierce, Thermo Scientific™). Then, pooled liver lysates (20 µg) from three individual samples were separated by 8% gel in SDS-PAGE and transferred to nitrocellulose membranes (Trans-Blot, Bio-Rad, Hercules, CA, USA). Western blotting was performed as previously described using Cyp2E1 (Abcam, Waltham, MA, USA) and β-actin (Cell Signaling, Danvers, MA, USA) antibodies [26]. Bands were visualized with enhanced chemiluminescence (Bio-Rad, Hercules, CA, USA).

### 2.9. Biliary Bile Acid Analysis

Biles were collected using syringe with 30 G needles and stored at −80 °C. 2 µL of bile were subjected to Metabolic Phenotyping Core at UTSW for bile acids composition analysis by LC-MS/MS.

### 2.10. Cytokine Multiplex Assay

Plasma concentrations of MCP1, TNFα, CXCL1, MIP1α and IL6 were determined using the MILLIPLEX MAP Mouse Cytokine/Chemokine assay (Millipore, Burlington, MA, USA).

### 2.11. Statistical Analysis

Data are expressed as mean ± SEM. Differences between two groups were analyzed using an unpaired Student’s *t*-test (GraphPad Prism 10, Boston, MA, USA). *p* < 0.05 was considered statistically significant.

## 3. Results

### 3.1. Ezetimibe Attenuates Hepatic Triglyceride Accumulation Induced by Combined Feeding of Dietary Cholesterol and Alcohol in Mice

**Figure 1:**
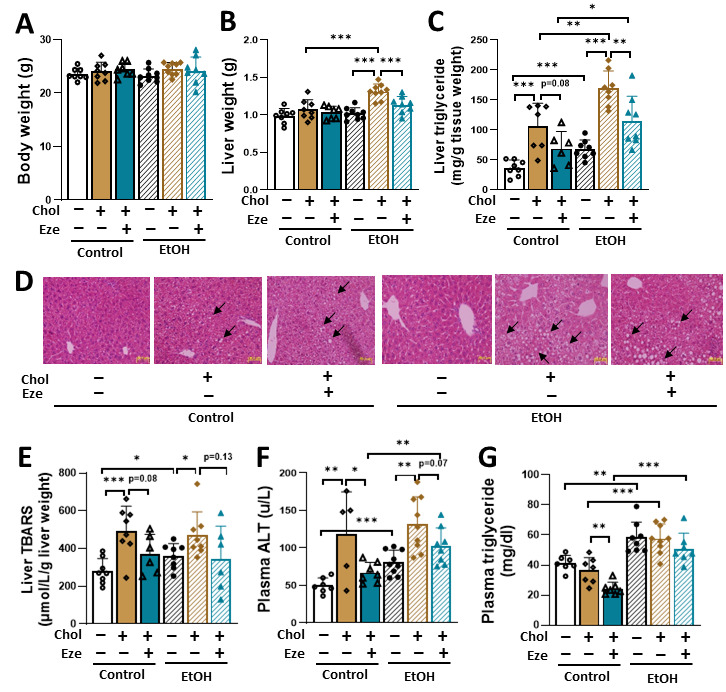
Dietary cholesterol exacerbates alcohol-induced hepatic steatosis, which is attenuated by ezetimibe. C57BL/6J male mice were fed one of the six liquid diets for 14 days followed by a single alcohol binge (EtOH, 5 g/kg BW) or isocaloric maltose dextrin (Control) on the 15th day. (**A**) Body weight. (**B**) Liver weight. (**C**) Liver triglyceride. (**D**) H&E staining of liver sections. Arrows indicate lipid droplets. Scale bar: 50µm. (**E**) Hepatic content of TBARS. (**F**) Plasma ALT. (**G**) Plasma triglyceride. *n* = 5–9. * *p* < 0.05, ** *p* < 0.01, *** *p* < 0.001. Abbreviations: Chol, cholesterol; Eze, ezetimibe; ALT, alanine aminotransferase; TBARS, thiobarbituric acid reactive substance.

To examine whether dietary cholesterol potentiates liver damage in mice exposed to NIAAA alcohol feeding model and whether the synergistic adverse effect can be reversed by ezetimibe, C57BL/6J male mice were subjected to six types of liquid diets as described in “Materials and Methods”. Neither dietary cholesterol nor ezetimibe affected body weight (Figure 1A). The liquid diet containing both EtOH and cholesterol (EtOH+Chol) caused a significant increase in liver weight, and this elevation was greatly attenuated by ezetimibe (EtOH+Chol+Eze: 1.13 ± 0.04 g versus EtOH+Chol: 1.31 ± 0.03 g, *p* < 0.001) (Figure 1B). In line with the role of alcohol in causing liver injury, relative to Control-fed mice, EtOH-fed mice showed significantly elevated hepatic triglyceride and plasma ALT (Figure 1C,F). The addition of dietary cholesterol further increased hepatic fat content and circulating ALT in both Control- and EtOH-treated mice (Figure 1C,F). Moreover, hepatic triglyceride was higher in mice fed EtOH+Chol compared to mice on Control+Chol diet. By blocking intestinal cholesterol absorption, ezetimibe significantly attenuated hepatic steatosis and plasma ALT in Control+Chol- and EtOH+Chol-fed mice, although values in the EtOH+Chol+Eze group remained higher than those in mice not exposed to ethanol (Figure 1C,F). Histological analysis further supported these findings, as H&E staining revealed extensive lipid droplet accumulation in the livers of EtOH+Chol-fed mice, which was substantially reduced by ezetimibe treatment (Figure 1D). Alcohol feeding significantly elevated hepatic thiobarbituric acid reactive substance (TBARS) contents (Figure 1E), and dietary cholesterol exacerbated this increase in both Control- and EtOH-fed mice. Interestingly, these elevations were slightly attenuated in Control+Chol+Eze- but not EtOH+Chol+Eze-fed mice. No significant changes were observed in plasma AST levels (Supplemental Figure S1A). It has been reported that alcohol drinking leads to elevated plasma triglyceride in rodents and human subjects [27,28]. Similar findings were observed in the current study (EtOH: 58.3 ± 3.7 mg/dL versus Control: 41.3 ± 1.9 mg/dL, *p* < 0.01) (Figure 1G). Dietary cholesterol itself did not affect circulating triglyceride (Figure 1G). Interestingly, ezetimibe treatment reduced plasma triglyceride in mice fed Control+Chol, but not EtOH+Chol liquid diet (Figure 1G). Alcohol has been shown to enhance adipose tissue lipolysis [27,29,30]. In line with this concept, significantly increased circulating non-esterified fatty acid (NEFA) was observed in EtOH-fed mice compared to Control-fed animals (Supplemental Figure S1B). Comparable NEFA levels among EtOH, EtOH+Chol, and EtOH+Chol+Eze groups indicated that neither dietary cholesterol nor ezetimibe influenced alcohol-induced adipocyte lipolytic function.

### 3.2. Ezetimibe Treatment Upregulates the Expression of Genes Involved in Hepatic Beta Oxidation

**Figure 2:**
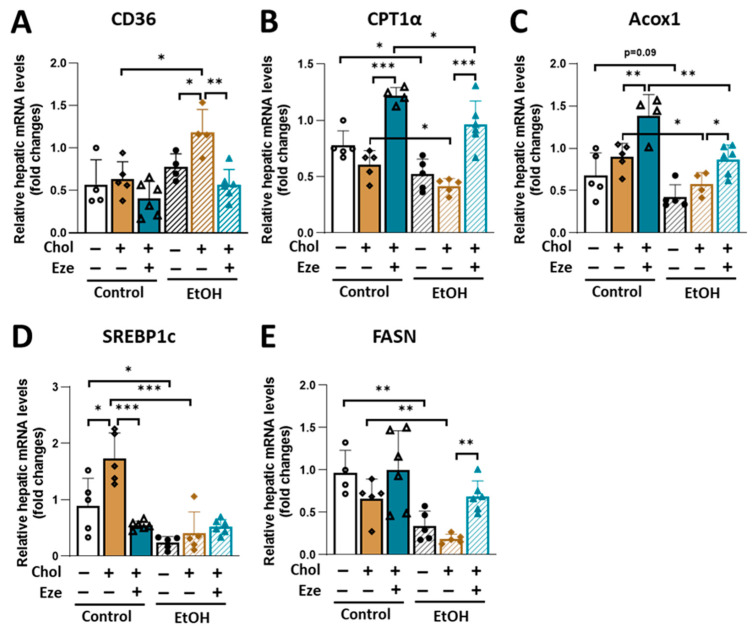
The effect of dietary cholesterol and ezetimibe on hepatic mRNA expression of genes involved in lipid metabolism in Control- and EtOH-fed mice. Relative hepatic mRNA levels of genes in (**A**) fatty acid transport (CD36), (**B**,**C**) fatty acid oxidation (CPT1α and Acox1), (**D**,**E**) fatty acid synthesis (SREBP1c and FASN). *n* = 4–6. * *p* < 0.05, ** *p* < 0.01, *** *p* < 0.001. Abbreviations: Chol, cholesterol; Eze, ezetimibe.

To determine how dietary cholesterol exacerbates alcohol-induced hepatic steatosis and liver injury, we examined hepatic expression of key genes involved in fatty acid transport and oxidation as well as de novo lipogenesis (DNL). As shown in Figure 2A, the addition of cholesterol to EtOH-containing diet caused a significant increase in CD36 expression in the liver, which was markedly blunted by ezetimibe. Consistent with the established role of alcohol in suppressing hepatic beta oxidation [31,32], expression of carnitine palmitoyltransferase 1α (CPT1α) and acyl-CoA oxidase 1 (Acox1) was significantly reduced in mouse livers exposed to EtOH and EtOH+Chol diets (Figure 2B,C). Although these two genes were not affected by dietary cholesterol, their expression levels were significantly increased by ezetimibe (Figure 2B,C). Several findings reported that acute-on-chronic alcohol feeding decreases hepatic expression of genes controlling DNL [27,33]. In agreement, greatly reduced mRNA levels of sterol regulatory element binding protein 1c (SREBP1c) and fatty acid synthase (FASN) were observed in the livers collected from mice fed EtOH-containing liquid diets regardless of dietary cholesterol (Figure 2D,E). We also observed that Control+Chol diet caused an increase in hepatic SREBP1c expression, which was suppressed by ezetimibe supplementation (Figure 2D). Ezetimibe enhanced FASN expression in the livers of mice fed EtOH+Chol diet (Figure 2E). Consistent with previous reports that alcohol feeding but not dietary cholesterol affects hepatic expression of Cyp2E1 [3], an enzyme responsible for alcohol metabolism, comparably increased Cyp2E1 expression was observed in the livers of mice fed EtOH, EtOH+Chol and EtOH+Chol+Eze diets (Supplemental Figure S1C).

### 3.3. Ezetimibe Prevents Dietary Cholesterol-Induced Hepatic Cholesterol Accumulation in Mice

**Figure 3:**
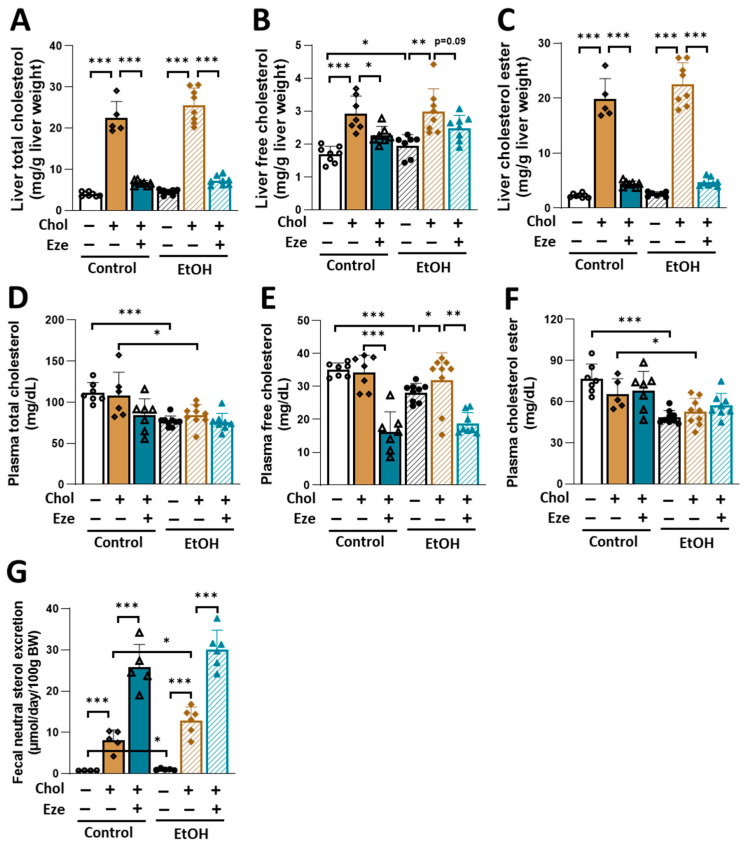
Ezetimibe normalizes dietary cholesterol-induced increases in hepatic cholesterol content in mice. (**A**) Liver total cholesterol. (**B**) Liver free cholesterol. (**C**) Liver cholesterol ester. (**D**) Plasma total cholesterol. (**E**) Plasma free cholesterol. (**F**) Plasma cholesterol ester. (**G**) Fecal neutral sterol excretion. *n* = 4–9. * *p* < 0.05, ** *p* < 0.01, *** *p* < 0.001. Abbreviations: Chol, cholesterol; Eze, ezetimibe.

Dietary cholesterol has profound effects on whole-body cholesterol metabolism. When 0.2% cholesterol was added to liquid diets, hepatic total cholesterol, free cholesterol and cholesterol ester were dramatically and comparably increased in both Control- and EtOH-fed mice (Figure 3A–C). However, dietary cholesterol did not affect plasma cholesterol levels (Figure 3D–F). Although hepatic free cholesterol content was slightly but significantly increased in mice following acute-on-chronic alcohol feeding (Figure 3B), circulating free cholesterol levels were decreased (Figure 3D–F). Regardless of dietary cholesterol, alcohol feeding reduced plasma total cholesterol and cholesterol ester in mice (Figure 3D,F). Ezetimibe treatment successfully rescued dietary cholesterol-induced dramatic elevations in hepatic cholesterol contents in Control- and EtOH-fed mice (Figure 3A–C). Although ezetimibe did not affect plasma total cholesterol and cholesterol ester concentrations (Figure 3D,F), it greatly reduced circulating free cholesterol levels (Figure 3E).

Alterations in cholesterol metabolism are closely associated with changes in fecal neutral sterol excretion [34,35]. However, it remains unknown how alcohol alone or in combination with dietary cholesterol affects fecal contents of neutral sterol. Compared to Control-fed mice, EtOH-fed mice exhibited significantly increased neutral sterol excretion (EtOH: 1.03 ± 0.10 µmol/day/100 g BW versus Control: 0.75 ± 0.03 µmol/day/100 g BW, *p* < 0.01) (Figure 3G). The consumption of dietary cholesterol led to markedly elevated excretions of fecal neutral sterols in both Control- and EtOH-fed mice. Furthermore, EtOH+Chol-fed mice showed higher fecal neutral sterol excretion than Control+Chol-fed mice. As shown in Figure 3G, when ezetimibe was added to cholesterol-containing diets, dramatically increased excretions of fecal neutral sterol were observed and the elevations were comparable between Control+Chol+Eze- and EtOH+Chol+Eze-fed mice.

### 3.4. Ezetimibe Reverses Dietary Cholesterol-Induced Changes in Genes Involved in Cholesterol Metabolism in Mice

**Figure 4:**
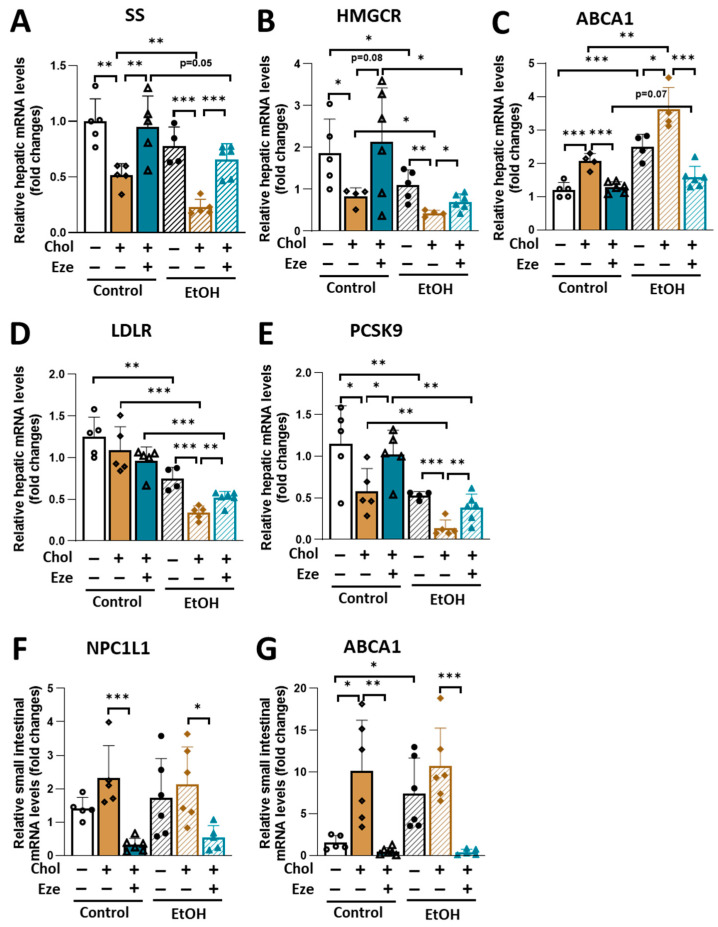
The impact of dietary cholesterol and ezetimibe on mRNA expression of genes involved in cholesterol metabolism in Control- and EtOH-fed mice. (**A**–**E**) Relative hepatic mRNA levels of genes in (**A**,**B**) cholesterol synthesis (SS and HMGCR), (**C**) cholesterol efflux (ABCA1), (**D**,**E**) cholesterol uptake (LDLR and PCSK9). (**F**,**G**) Relative mRNA levels of genes in intestinal (**F**) cholesterol uptake (NPC1L1) and (**G**) cholesterol efflux (ABCA1). *n* = 4–6. * *p* < 0.05, ** *p* < 0.01, *** *p* < 0.001. Abbreviations: Chol, cholesterol; Eze, ezetimibe.

The consumption of cholesterol leads to suppression of endogenous cholesterol synthesis [36]. Consistent with this concept, dramatically reduced hepatic mRNA levels of squalene synthase (SS) and 3-hydroxy-3-methylglutaryl-coenzyme A reductase (HMGCR), two key enzymes in de novo cholesterol synthesis, were observed in mice on cholesterol-containing diets (Figure 4A,B). In addition, these suppressions were more dramatic in EtOH+Chol-fed mice than in Control+Chol-treated animals. By inhibiting intestinal cholesterol absorption, ezetimibe completely rescued cholesterol-induced reductions in the expression of SS and HMGCR in the liver. In addition, the expression of these two enzymes was lower in EtOH+Chol+Eze mice than in Con+Chol+Eze mice (Figure 4A,B). Greatly elevated mRNA levels of ATP-binding cassette sub-family A member 1 (ABCA1) were found in mice exposed to dietary cholesterol but dramatically blunted by ezetimibe supplementation (Figure 4C). Relative to Control-fed mice, alcohol overconsumption caused an increase in hepatic ABCA1 expression in mice (Figure 4C). Regardless of dietary cholesterol and ezetimibe, acute-on-chronic alcohol feeding greatly suppressed hepatic expression of low-density lipoprotein receptor (LDLR) and proprotein convertase subtilisin/kexin type 9 (PCSK9), two important genes responsible for hepatic and systemic cholesterol metabolism (Figure 4D,E). Cholesterol caused a decrease in hepatic LDLR expression in EtOH-fed mice (Figure 4D). Reduced PCSK9 expression in the livers of both Control+Chol- and EtOH+Chol-fed mice was observed (Figure 4E). Of note, these reductions were restored by ezetimibe (Figure 4D,E).

NPC1L1 is a transmembrane protein in the proximal small intestine and plays a critical role in cholesterol absorption [37]. Neither alcohol nor dietary cholesterol affected intestinal NPC1L1 expression (Figure 4F). Interestingly, intestinal NPC1L1 expression was greatly reduced in mice on Control+Chol+Eze and EtOH+Chol+Eze liquid diets. Intestinal ABCA1 is an important regulator for maintaining intestinal cholesterol balance. Compared to Control-fed mice, EtOH-fed mice showed significantly elevated ABCA1 expression in small intestine (Figure 4G). Dietary cholesterol-induced elevations in ABCA1 expression in mouse intestine were dramatically suppressed by the presence of ezetimibe (Figure 4G).

**Figure 5:**
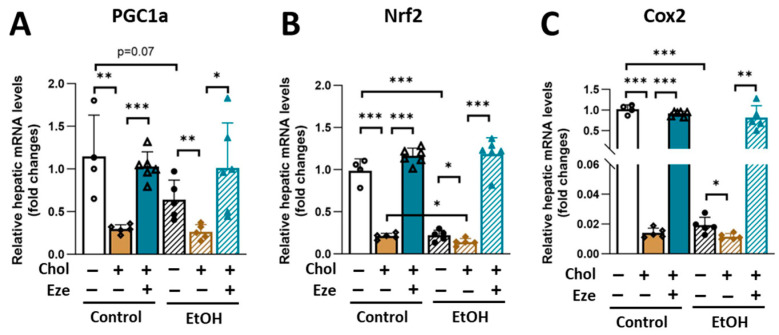
Ezetimibe normalizes the suppressive effect of dietary cholesterol on hepatic mRNA expression of PGC1α, Nrf2 and Cox2 in Control- and EtOH-fed mice. qPCR analysis of (**A**) PGC1α, (**B**) Nrf2, and (**C**) Cox2. *n* = 4–6. * *p* < 0.05, ** *p* < 0.01, *** *p* < 0.001. Abbreviations: Chol, cholesterol; Eze, ezetimibe.

Dietary cholesterol has been shown to increase mitochondrial cholesterol content and disrupt mitochondrial function [38,39,40,41]. In contrast, ezetimibe treatment attenuates mitochondrial dysfunction in various cell types, including polymorphonuclear leukocytes [42], human umbilical vein endothelial cells [43], and cardiac myocytes [44]. Based on these findings, we sought to determine hepatic expression levels of genes involved in mitochondrial function, including peroxisome proliferator-activated receptor gamma coactivator 1-alpha (PGC1α), nuclear factor erythroid 2-related factor 2 (Nrf2) and cytochrome c oxidase II (Cox2). We found that hepatic expression of all three genes was substantially suppressed by dietary cholesterol but was completely restored by ezetimibe treatment in both Control- and EtOH-fed mice (Figure 5A–C). In agreement with the established role of excessive alcohol consumption in impairing mitochondrial function [45], reduced hepatic expression of PGC1α, Nrf2 and Cox2 was observed in EtOH-fed mice (Figure 5A–C). Notably, supplementation of dietary cholesterol further suppressed the expression of these genes, indicating a synergistic effect of alcohol and cholesterol on mitochondrial dysfunction.

### 3.5. Regulation of Gallbladder Bile Acids by Ezetimibe in Mice Exposed to Liquid Diets Containing Alcohol and/or Dietary Cholesterol

**Figure 6:**
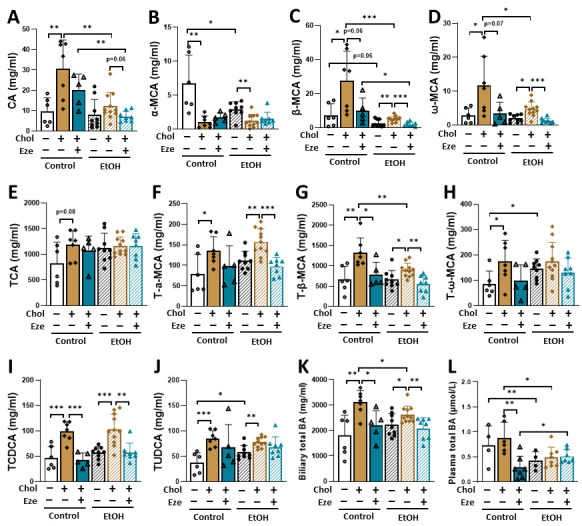
Biliary bile acid composition in mice fed various diets. Gallbladder bile was collected and bile acid composition was quantified by LC-MS/MS. (**A**) CA. (**B**) α-MCA. (**C**) β-MCA. (**D**) ω-MCA. (**E**) TCA. (**F**) T-α-MCA. (**G**) T-β-MCA. (**H**) T-ω-MCA. (**I**) TCDCA. (**J**) TUDCA. (**K**) Biliary total BA. (**L**) Plasma total BA. *n* = 4–11. * *p* < 0.05, ** *p* < 0.01, *** *p* < 0.001. Abbreviations: Chol, cholesterol; Eze, ezetimibe; CA, cholic acid; α-MCA, α-muricholic acid; β-MCA, β-muricholic acid; ω-MCA, ω-muricholic acid; TCA, taurocholic acid; T-α-MCA, Tauro-α-muricholic acid; T-β-MCA, Tauro-β-muricholic acid; T-ω-MCA, Tauro-ω-muricholic acid; TCDCA, taurochenodeoxycholic acid; TUDCA, tauroursodeoxycholic acid; BA, bile acid.

One of the cholesterol catabolic pathways in the liver is to synthesize bile acids and transport them to gallbladder. Compared to mice fed a Control-only diet, mice on Control+Chol diet exhibited increased biliary contents of cholic acid (CA), β-muricholic acid (β-MCA) and ω-MCA (Figure 6A,C,D). Combined feeding of cholesterol and alcohol (EtOH+Chol) slightly but significantly increased β-MCA and ω-MCA in mouse gallbladder compared to EtOH only group (Figure 6C,D). These increases were greatly blunted by ezetimibe treatment (Figure 6C,D). Interestingly, biliary α-MCA contents were significantly reduced by dietary cholesterol in both Control- and EtOH-fed mice although α-MCA represented only a minor fraction of total bile acids in the gallbladder (Figure 6B). For taurine-conjugated bile acids, dietary cholesterol enhanced biliary taurocholic acid (TCA) and T-ω-MCA levels in Control-, but not EtOH-fed mice (Figure 6E,H). Interestingly, elevated T-α-MCA, T-β-MCA, taurochenodeoxycholic acid (TCDCA) and tauroursodeoxycholic acid (TUDCA) contents were observed in the gallbladder of mice fed either Control+Chol or EtOH+Chol diet (Figure 6F,G,I,J). Except for TUDCA, these cholesterol-induced elevations were markedly reversed by ezetimibe treatment (Figure 6F–I). In addition, alcohol alone increased biliary T-ω-MCA and TUDCA levels (Figure 6H,J) whole suppressing unconjugated α-MCA and β-MCA contents (Figure 6B,C). Compared to other taurine-conjugated bile acids, taurodeoxycholic acid (TDCA) and taurohyodeoxycholic acid (THDCA) were present at relatively low levels in the gallbladder and were markedly reduced in EtOH+Chol+Eze-treated mice (Supplemental Figure S2A,B). Three glycine-conjugated bile acids, glycocholic acid (GCA), glycochenodeoxycholic acid (GCDCA) and glycoursodeoxycholic acid (GUDCA) were detected at trace levels in mouse gallbladders (Supplemental Figure S2C–E). These bile acids were increased following alcohol exposure. Moreover, Control+Chol feeding-induced elevations in these three bile acids were blunted by ezetimibe treatment (Supplemental Figure S2C–E). Figure 6K showed the effects of alcohol feeding and dietary cholesterol on total biliary bile acids in mice. Alcohol alone did not affect total bile acid content in mouse gallbladder. Regardless of the treatment (Control or EtOH), dietary cholesterol significantly increased biliary total bile acids in mice and these elevations were greatly attenuated by ezetimibe treatment. In contrast, alcohol overconsumption decreased plasma total bile acid (Figure 6L). Ezetimibe treatment lowered plasma bile acid levels in Control+Chol-, but not EtOH+Chol-fed mice (Figure 6L).

**Figure 7:**
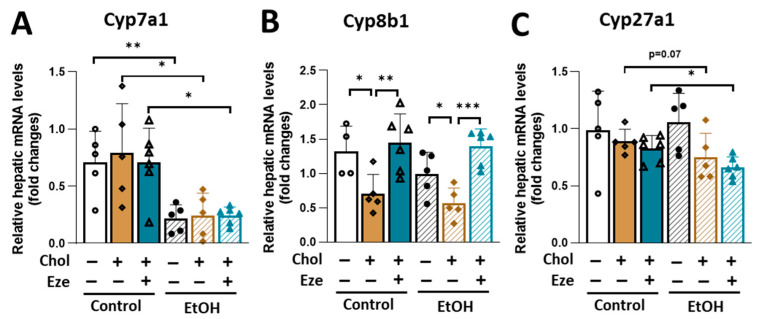
The effect of dietary cholesterol and ezetimibe on hepatic mRNA expression of genes involved in bile acid biosynthesis in Control- and EtOH-fed mice. Relative hepatic mRNA levels of genes in bile acid synthesis. (**A**) Cyp7a1. (**B**) Cyp8b1. (**C**) Cyp27a1. *n* = 4–6. * *p* < 0.05, ** *p* < 0.01, *** *p* < 0.001. Abbreviations: Chol, cholesterol; Eze, ezetimibe.

Cytochrome P450 family 7 sub-family A member 1 (Cyp7a1) is the rate-limiting enzyme in classic bile acid synthetic pathway. Its hepatic expression was suppressed by alcohol feeding regardless of dietary cholesterol or ezetimibe (Figure 7A). The expression of Cyp8b1 was significantly reduced by cholesterol feeding in both Control- and EtOH-fed mice, and this suppression was reversed by ezetimibe treatment (Figure 7B). Relative to Control+Chol+Eze-fed mice, Cyp27a1 expression was significantly lower in the livers of EtOH+Chol+Eze-fed mice (Figure 7C).

### 3.6. The Effects of Ezetimibe on Tissue and Systemic Inflammation in Mice Fed Liquid Diets Containing Alcohol and/or Dietary Cholesterol

**Figure 8:**
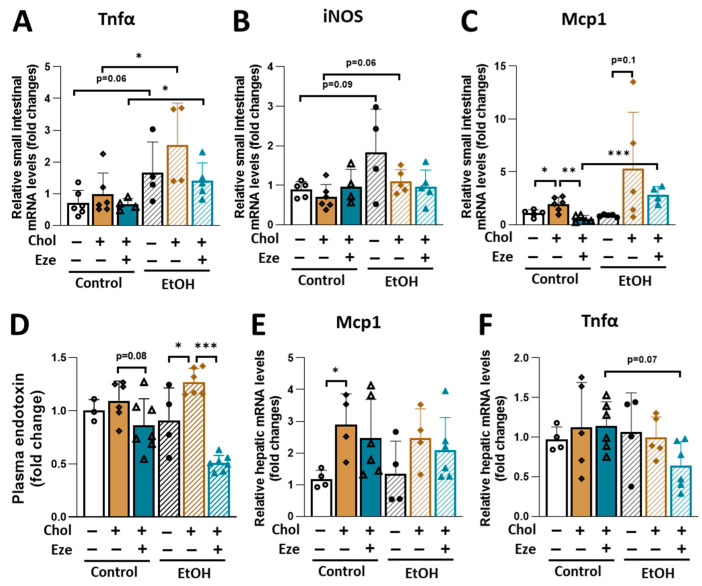
The impact of dietary cholesterol and ezetimibe on mRNA expression of genes involved in inflammation in Control- and EtOH-fed mice. (**A**–**C**) Relative intestinal mRNA levels of genes in inflammation (**A**) Tnfα, (**B**) iNOS and (**C**) Mcp1. (**D**) Plasma endotoxin (fold changes). (**E**,**F**) Relative hepatic mRNA levels of genes in inflammation (**E**) Mcp1 and (**F**) Tnfα. *n* = 3–6. * *p* < 0.05, ** *p* < 0.01, *** *p* < 0.001. Abbreviations: Chol, cholesterol; Eze, ezetimibe.

Alcohol can impair epithelial tight junctions by increasing intestinal pro-inflammatory response [46,47,48] and activating inducible nitric oxide synthases (iNOS) [49,50]. In line with this concept, we observed that alcohol feeding alone elevated tumor necrosis factor alpha (Tnfα) and iNOS expression in the small intestine (Figure 8A,B). Dietary cholesterol has been shown to promote intestinal inflammation [51]. Consistent with these findings, the cholesterol supplementation greatly increased intestinal monocyte chemoattractant protein 1 (Mcp1) expression in Control-fed mice, which was blunted by ezetimibe treatment (Figure 8C). Moreover, compared to mice exposed to Control+Chol+Eze diet, mice fed EtOH+Chol+Eze exhibited significantly higher expression of intestinal Tnfα and Mcp1 (Figure 8A,C). Figure 8D showed the fold changes in plasma endotoxin across different dietary groups. Dietary cholesterol promoted plasma endotoxin levels in EtOH-fed mice, an effect that was greatly reduced by ezetimibe. Compared to the Control+Chol group, mice on Control+Chol+Eze diet exhibited slightly lower plasma endotoxin concentration. Dietary cholesterol tended to increase hepatic Mcp1 expression in both Control- and EtOH-fed mice (Figure 8E). Although hepatic Tnfα expression was not affected by either dietary cholesterol or alcohol feeding, its expression was slightly lower in mice fed EtOH+Chol+Eze diet compared to those on Control+Chol+Eze diet (Figure 8F).

**Figure 9:**
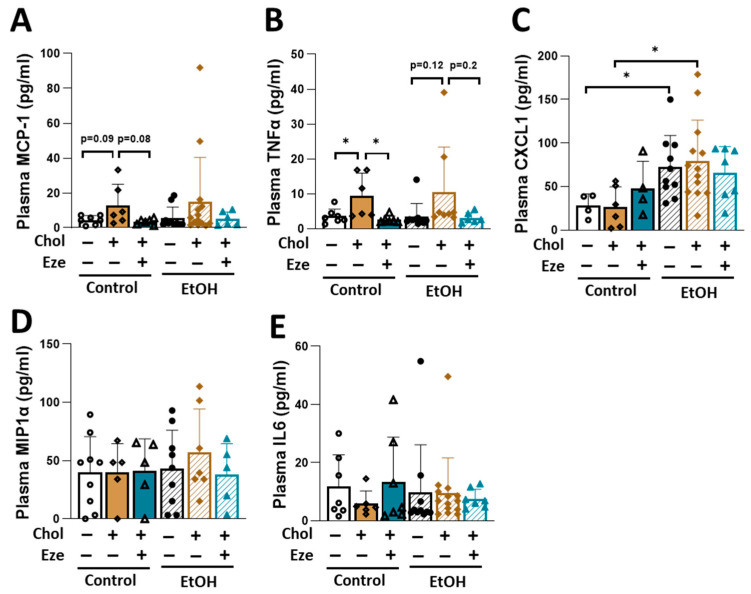
Circulating inflammatory cytokines in mice fed various diets. Plasma concentrations of inflammatory cytokines. (**A**) MCP1. (**B**) TNFα. (**C**) CXCL1. (**D**) MIP1α. (**E**) IL6. *n* = 4–13. * *p* < 0.05. Abbreviations: Chol, cholesterol; Eze, ezetimibe.

Analysis of circulating inflammatory cytokines showed that plasma MCP1 tended to increase by dietary cholesterol in Control-fed mice, an effect that was reversed by ezetimibe treatment (Figure 9A). In addition, cholesterol-induced increases in plasma TNFα levels in both Control- and EtOH-fed mice were blunted by ezetimibe (Figure 9B). Chemokine (C-X-C motif) ligand 1 (CXCL1) expression is highly upregulated in livers of subjects with alcoholic hepatitis [52], and CXCL1 deficiency attenuates high fat diet plus binge ethanol-induced liver injury in mice [53]. In the current study, we found that both alcohol alone and combined feeding of alcohol and cholesterol led to elevated circulating CXCL1 levels (Figure 9C). Plasma macrophage inflammatory protein 1 (MIP1α) and IL6 levels were not significantly altered by alcohol or dietary cholesterol (Figure 9D,E).

## 4. Discussion

Multiple risk factors have been implicated in the development and progression of ALD, such as genetic background, gender, smoking and nutritional conditions [2,54]. A recent cross-sectional study reported by Tanisawa et al. showed that subjects who consume excessive amounts of alcohol tend to have higher dietary cholesterol intake [55]. Of note, diets rich in high cholesterol are quite common in Western societies [18]. Both clinical studies and experimental animal models have reported that dietary cholesterol leads to progressive liver damage [56]. Therefore, it is important to investigate whether cholesterol potentiates alcohol-induced liver injury and whether blocking its absorption can reverse the liver damage caused by combined alcohol and cholesterol consumption.

Alcohol feeding has been shown to increase hepatic and circulating cholesterol contents [57,58,59], probably resulting from increased hepatic cholesterol synthesis [57,58]. Interestingly, in the current study, alcohol only liquid diet did not cause dramatic changes in hepatic cholesterol content but had profound effects on the expression of genes involved in cholesterol metabolism, including significantly reduced HMGCR and LDLR as well as increased ABCA1 in the liver. These alcohol-induced changes in genes associated with hepatic cholesterol synthesis, uptake and high-density lipoprotein (HDL) formation have been reported previously [11,60]. In contrast to prior reports showing the development of hypercholesterolemia in rodents following excessive alcohol consumption [58,59], we found significant decreases in plasma cholesterol levels in mice following acute-on-chronic alcohol exposure. In support of our findings, Kong et al. also observed decreased serum levels of total cholesterol in C57BL/6 male mice after NIAAA alcohol feeding [33]. The discrepancies regarding the effect of alcohol on cholesterol metabolism are not clear and could be due to differences in experimental models (in vivo versus ex vivo), patterns of alcohol exposure (chronic versus acute-on-chronic feeding), and variations in species (mice versus rats).

Krishnasamy et al. reported that chronic feeding of combined diets containing alcohol (5%) and 0.5% (*w*/*w*) cholesterol for 3 months leads to the greatest hepatic cholesterol content in mice [3]. In the present study, we observed that dietary cholesterol resulted in dramatic increases in liver cholesterol accumulation, which was comparable between mice fed Control+Chol and EtOH+Chol diets following the acute-on-chronic feeding model. We also found that EtOH+Chol diet caused the lowest expression of SS, HMGCR and LDLR as well as the highest expression of ABCA1 in mouse livers. These findings indicate that combined EtOH+Chol feeding did not further elevate hepatic cholesterol levels, likely due to suppressed hepatic cholesterol synthesis and uptake as well as enhanced cholesterol excretion.

Ezetimibe has been shown to effectively normalize liver and systemic cholesterol dysregulation [13,61]. Consistently, our data showed that dietary cholesterol-induced elevations in hepatic cholesterol and alterations in genes involved in cholesterol metabolism were reversed by ezetimibe treatment in both Control- and EtOH-fed mice. In agreement with prior reports that ezetimibe enhances fecal neutral sterol excretion in mice [62], we found that Control+Chol+Eze- and EtOH+Chol+Eze-fed mice exhibited the highest fecal neutral sterol excretion.

Dietary cholesterol has been shown to worsen liver injury in mice fed alcohol chronically, indicated by great increases in hepatic triglycerides and circulating ALT levels [3,4]. Similar findings were observed in the current study using an acute-on-chronic feeding model with liquid diets supplemented with 0.2% Chol. Dietary cholesterol caused hepatic fat accumulation and liver injury in both Control- and EtOH-fed mice. Compared to mice on Control+Chol diet, mice consuming EtOH+Chol diet exhibited higher hepatic triglyceride contents, accompanied by further reductions in CPT1α and Acox1 expression. Different from a prior report showing that addition of 0.5% cholesterol alters CPT1α and FASN expression in mouse livers [3], we found that 0.2% dietary cholesterol alone had no impact on hepatic expression of CPT1α, Acox1 or FASN. Both clinical trials and preclinical experiments have demonstrated that ezetimibe treatment alleviates obesity-associated fatty liver disease [16,17,63,64,65,66]. Similarly, we found that dietary cholesterol-induced elevations in hepatic triglycerides and plasma ALT were greatly attenuated in both Control+Chol+Eze- and EtOH+Chol+Eze-fed mice. In addition, the expression of CPT1a and Acox1 was significantly increased in livers of mice exposed to ezetimibe-containing diets compared to their corresponding Control+Chol and EtOH+Chol groups. While alcohol or cholesterol alone did not affect hepatic CD36 expression, their combined intake markedly upregulated CD36 levels, an effect that was completely reversed by ezetimibe treatment, indicating the protective role of ezetimibe in suppressing hepatic lipid uptake.

Excessive alcohol intake impairs bile acid homeostasis. Elevated plasma total bile acids have been reported in patients with alcoholic hepatitis [67], which is associated with dramatically suppressed hepatic Cyp7a1 expression. Similarly, mice chronically administered 50% alcohol via oral gavage, but not 30%, exhibit significantly elevated plasma total bile acid despite dramatically elevated hepatic expression of genes involved in classic and alternative bile acid synthesis [12]. Interestingly, comparable or even reduced serum bile acids have been observed in mice exposed to chronic-plus-binge alcohol feeding, which is accompanied by decreased Cyp7a1 and Cyp8b1 expression in mouse livers [11,33,60]. Consistent with these reports, we found that alcohol feeding led to significant reductions in plasma bile acids and hepatic Cyp7a1 expression. These discrepancies are likely attributable to variations in animal species and alcohol feeding regimens. In terms of the effect of cholesterol on bile acid synthetic genes, Henkel et al. found that dietary cholesterol regulates hepatic Cyp7a1 expression in a time-dependent manner in mice [68]. Acute cholesterol feeding (7 days) increases, while chronic cholesterol feeding (12 weeks) suppresses hepatic Cyp7a1 expression. In the current study, we did not observe the alterations in Cyp7a1 or Cyp27a1 expression by dietary cholesterol. Interestingly, hepatic expression of Cyp8b1 was significantly suppressed in response to cholesterol feeding, an effect that was fully reversed upon ezetimibe administration.

Limited studies reported the impact of alcohol intake on biliary bile acid content and composition. Gong et al. found significantly elevated biliary total bile acid in mice following chronic (6 weeks) oral gavage of alcohol [12]. In particular, unconjugated bile acids (CA, α-MCA, β-MCA, and ω-MCA), taurine-conjugated bile acids (T-αMCA, T-βMCA, T-DCA, T-CDCA, T-HDCA, TUDCA, and T-LCA) and glycine-conjugated bile acids (GCA and GDCA) were all significantly elevated in alcohol-exposed mice. In contrast, Montet et al. reported that chronic feeding of alcohol-containing liquid diet did not affect bile acid contents in rats [69]. In line with the latter study, we also observed comparable amounts of total gallbladder bile acids in Control- and EtOH-treated mice following an acute-on-chronic feeding regimen. However, alterations in bile acid composition were observed, marked by suppressed biliary α-MCA and β-MCA and enhanced T-ωMCA, TUDCA, and glycine-conjugated bile acids (GCA, GCDCA and GUDCA). Dietary cholesterol has been shown to promote the accumulation of bile acid in the gallbladder [10]. Consistent with these findings, nearly all bile acids detected in the present study were elevated by dietary cholesterol supplementation in both Control- and EtOH-containing diets. Notably, these cholesterol-induced increases in biliary bile acid content were blunted by ezetimibe treatment.

**Figure 10:**
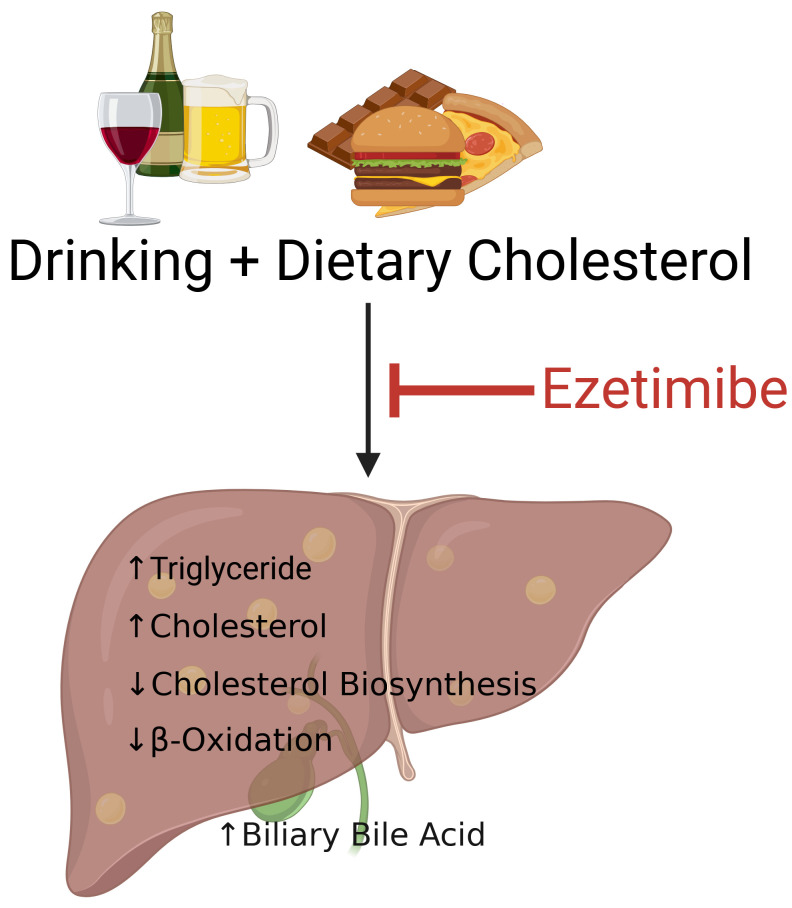
Diagram showing the essential role of ezetimibe in protecting against liver injury caused by heavy alcohol consumption in the setting of a high cholesterol diet. Figure was created with *BioRender*.

In summary, we found that mice consuming a diet containing both cholesterol and alcohol displayed the greatest increases in liver weight, hepatic triglyceride content and plasma ALT, effects that were markedly mitigated by ezetimibe treatment. Although hepatic cholesterol levels were comparably elevated in mice fed Control+Chol and EtOH+Chol diets, the livers of mice on the EtOH+Chol diet exhibited reduced expression of genes involved in cholesterol biosynthesis and increased expression of genes responsible for cholesterol efflux, relative to those on the Control+Chol diet. In addition, dietary cholesterol-induced increases in liver cholesterol content and gallbladder total bile acid were greatly attenuated by ezetimibe treatment (Figure 10). We also observed elevated inflammation in the small intestine and circulation in mice fed cholesterol-containing diet, which tended to be reduced following ezetimibe administration. These findings highlight the important role of ezetimibe in protecting against liver damage induced by heavy drinking in the context of a cholesterol-rich diet. Future studies will determine whether ezetimibe treatment can reverse liver injury and restore biliary bile acid homeostasis in mice pre-exposed to a combined diet containing both alcohol and cholesterol. These studies will further clarify the therapeutic potential of ezetimibe for treating liver injury resulting from excessive alcohol consumption and high cholesterol intake.

## 5. Conclusions

Ezetimibe reverses dietary cholesterol-driven worsening of liver injury in mice consuming excessive alcohol.

## Data Availability

The original contributions presented in this study are included in the article/Supplementary Material. Further inquiries can be directed to the corresponding author(s).

## Supplementary Materials

The following supporting information can be downloaded at: https://www.mdpi.com/article/10.3390/biom16040590/s1, Figure S1: Dietary cholesterol or ezetimibe did not affect plasma NEFA or hepatic Cyp2E1 expression in mice; Figure S2: Ezetimibe attenuates dietary cholesterol-induced increases in several biliary glycine-conjugated bile acids in Control- and EtOH-fed mice. Unedited blot images for Cyp2E1 and β-actin.
