# Supplementary material for: Ezetimibe Normalizes Dietary Cholesterol-Induced Exacerbation of Liver Injury in Alcohol-Fed Mice
Source: Biomolecules. 2026 Apr 16;16(4):590. doi: 10.3390/biom16040590 (PMC13113872; doi:10.3390/biom16040590)
Supplement: Supplementary file 1 [file biomolecules-16-00590-s001.zip › biomolecules-4191815 supplementary.pdf]

Ezetimibe Normalizes Dietary Cholesterol-induced Exacerbation of Liver Injury in Alcohol-fed Mice

Supplementary figures

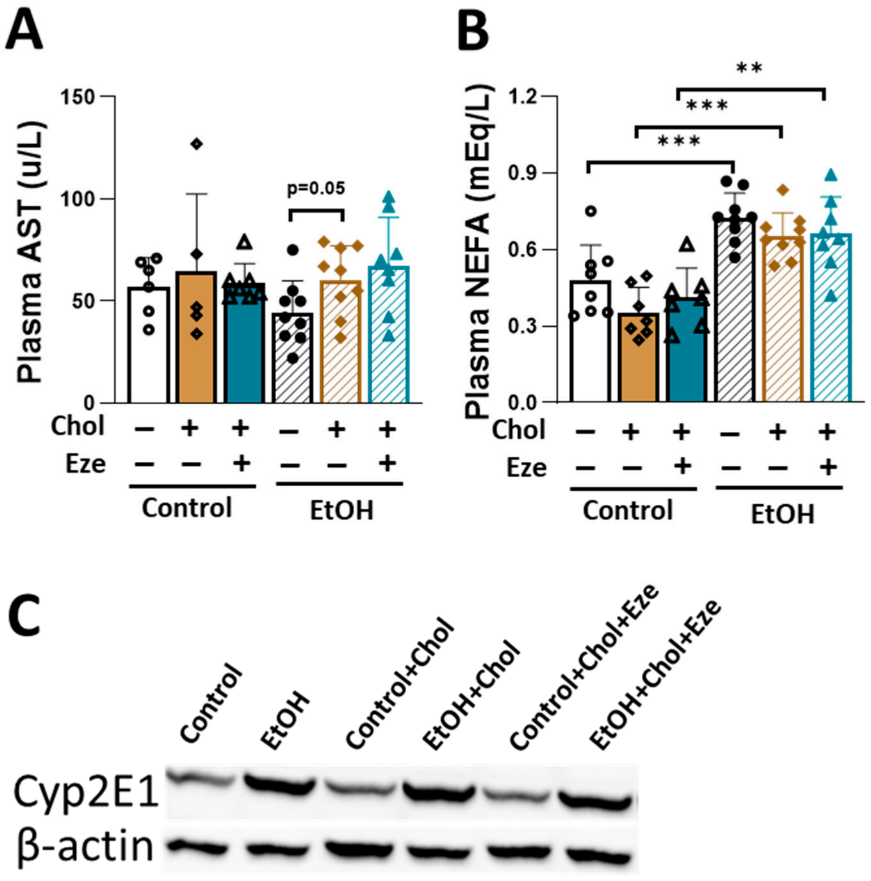

Supplemental Figure S1. Dietary cholesterol or ezetimibe did not affect plasma NEFA or hepatic Cyp2E1 expression in mice. (**A**) Plasma AST. (**B**) Plasma NEFA. (**C**) Hepatic protein expression of Cyp2E1. n = 5-9. *$p < 0.05$, **$p < 0.01$, *** $p < 0.001$. Abbreviations: Chol, cholesterol; Eze, ezetimibe; AST, aspartate aminotransferase; NEFA, non-esterified fatty acid. The original WB images can be found in the supplementary materials.

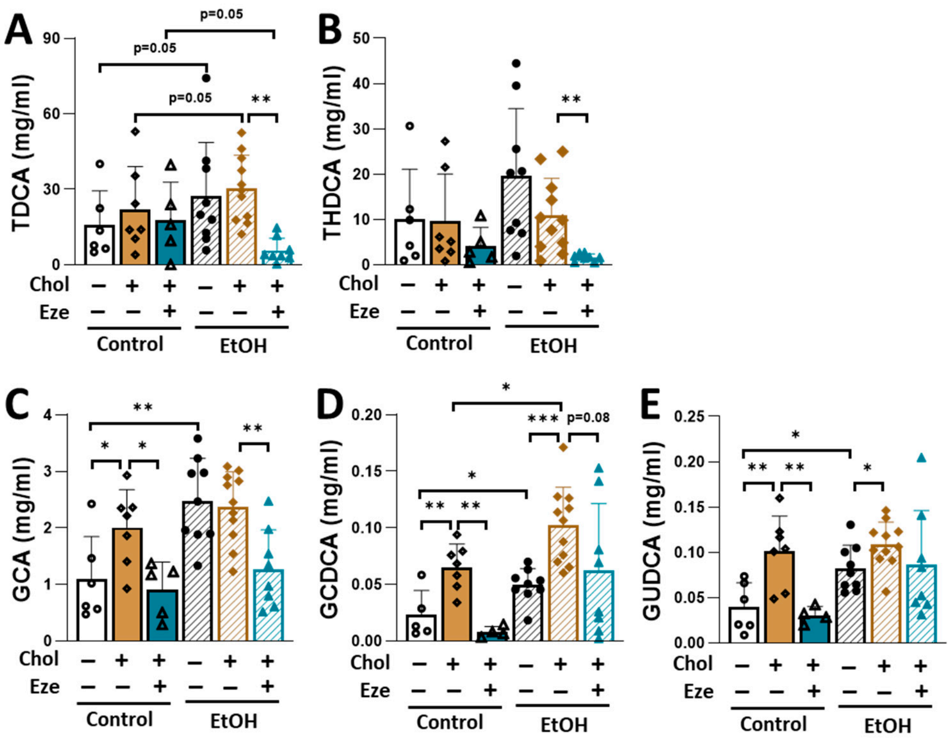

Supplemental Figure S2. Ezetimibe attenuates dietary cholesterol-induced increases in several biliary glycine-conjugated bile acids in Control- and EtOH-fed mice. Gallbladder bile acids were collected and quantified. (**A**) TDCA. (**B**) THDCA. (**C**) GCA. (**D**) GCDCA. (**E**) GUDCA. n = 4-11. *p < 0.05, **p < 0.01, *** p < 0.001. Abbreviations: Chol, cholesterol; Eze, ezetimibe; TDCA, taurodeoxycholic acid; THDCA, taurohyodeoxycholic acid; GCA, glycocholic acid; GCDCA, glycochenodeoxycholic acid; GUDCA, glycoursodeoxycholic acid.
