# Supplementary figures and images for: Ezetimibe Normalizes Dietary Cholesterol-Induced Exacerbation of Liver Injury in Alcohol-Fed Mice
Source: Biomolecules. 2026 Apr 16;16(4):590. doi: 10.3390/biom16040590 (PMC13113872; doi:10.3390/biom16040590)

Unedited blot images for Cyp2E1 and  $\beta$ -actin.

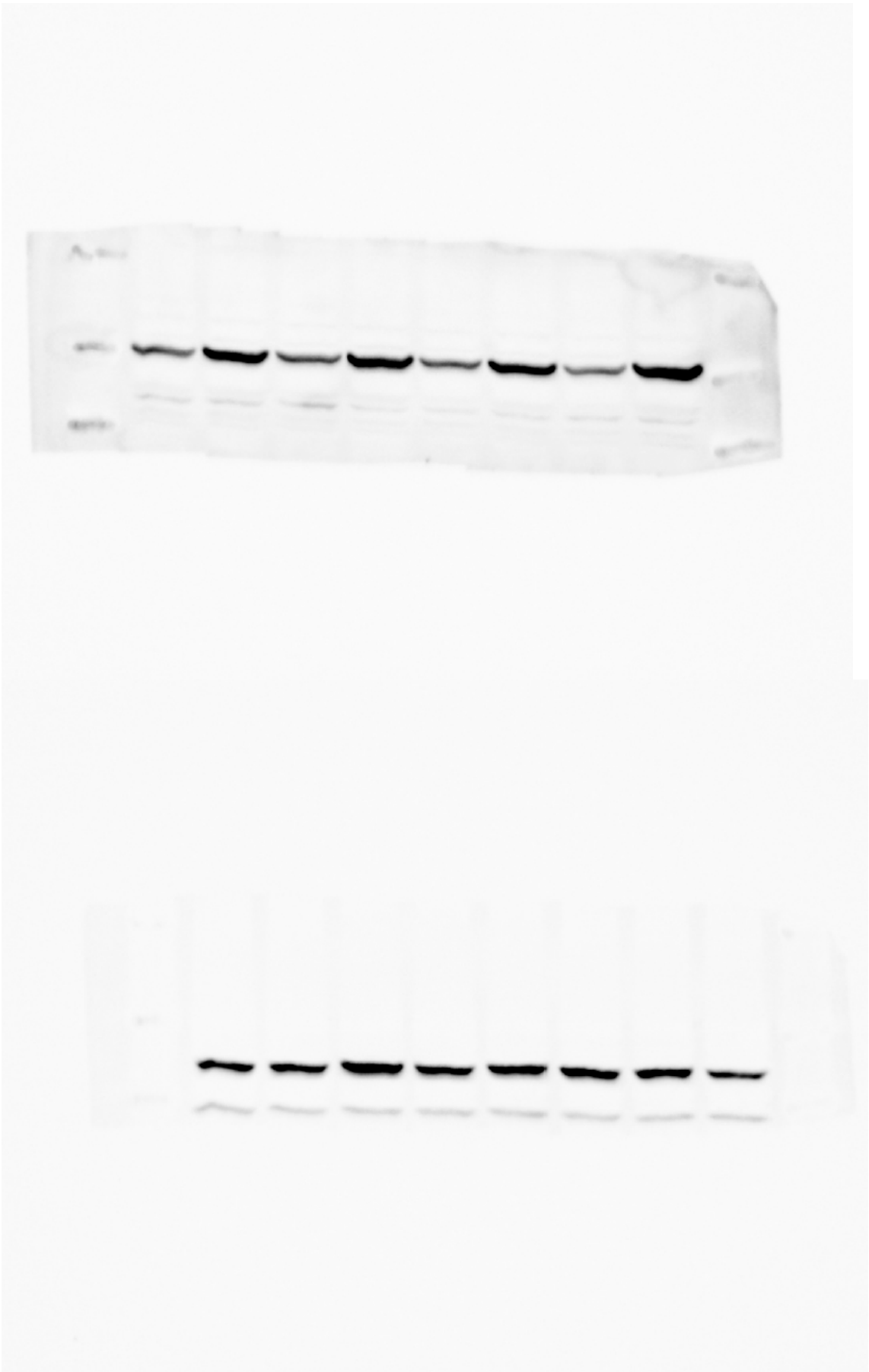

Supplement: Supplementary file 1 [file biomolecules-16-00590-s001.zip › biomolecules-4191815 original WB images.pdf]
